# An Esophagogram or Tracheobronchogram? A Review of Barium Sulfate Aspiration

**DOI:** 10.1177/2324709618802872

**Published:** 2018-10-04

**Authors:** Mohsin Hamid, Waqas Ullah, Mamoon Ur Rashid, Waseem Amjad, Maryam Mukhtar, Abu Hurairah

**Affiliations:** 1 Abington Memorial Hospital, Abington, PA, USA; 2 Florida Hospital, Orlando, FL, USA; 3 Northwell Health, Great Neck, NY, USA; 4 Fauji Foundation Hospital, Rawalpindi, Pakistan; 5 State University of New York Downstate Medical Center, Brooklyn, NY, USA

**Keywords:** barium aspiration, barium swallow

## Abstract

The barium swallow is an important radiological investigation used for the diagnosis of upper gastrointestinal anatomical disorders like esophageal cancer, diverticulum, achalasia, foreign body, among others. Generally, it is believed to be a safe technique with rare complications, but few cases of barium sulfate aspiration have been reported in the literature with multiple complications. We are reporting a case of an elderly male who underwent esophagogram for the workup of chronic dysphagia, aspirated barium sulfate, and went into respiratory failure and circulatory shock several hours later. Moreover, we also did a systematic literature search and reviewed all available articles on aspiration of barium sulfate and its potential complications. We focused on predisposing factors for aspiration, clinical presentation, complications after aspiration, and prognosis with the aim to better understand and manage this condition.

## Materials and Methods

An informed patient consent was obtained by the authors. The authors were actively involved in patient care and data were collected from the electronic medical records of the hospital.

## Case Presentation

An 84-year-old male with multiple comorbidities including prior ischemic stroke without any residual deficits was admitted to the hospital for worsening lethargy and weakness due to progressive dysphagia to solids and liquids and weight loss of 15 pounds in the past 6 to 8 months. According to the family, he had a long-standing history of coughing and choking while eating and complained about food getting stuck in his chest. His vitals were stable in the emergency department and was breathing on ambient air. Esophagogram was ordered for the workup of his chronic dysphagia. During esophagogram, he started coughing and choking after which the study was terminated. After 5 hours of study, he started becoming hypoxic with oxygen saturation of 86% on room air requiring a non-rebreather mask, fever of 101°F, tachycardia 112/min, and hypotension to 90s mm Hg systolic blood pressure. Aggressive intravenous (IV) resuscitation was done, IV antibiotics including vancomycin and piperacillin-tazobactam were started, and he was upgraded to intensive care unit for a higher level of care.

**Figure 1.**
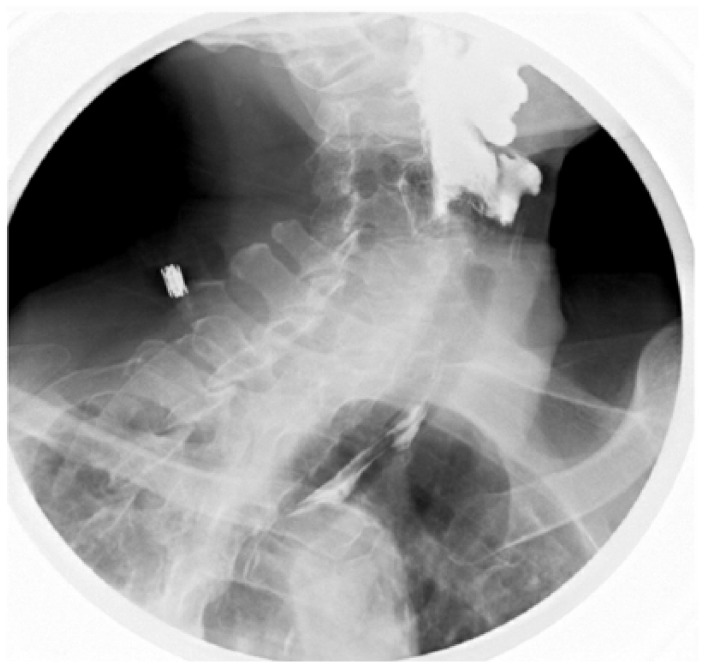
Early phase of aspiration into respiratory tract, that is, larynx and trachea.

Esophagogram showed early laryngeal penetration of contrast and subsequent presence of contrast in the trachea and bronchial tree with minimal contrast in the esophagus (Figure 1).

**Figure 2.**
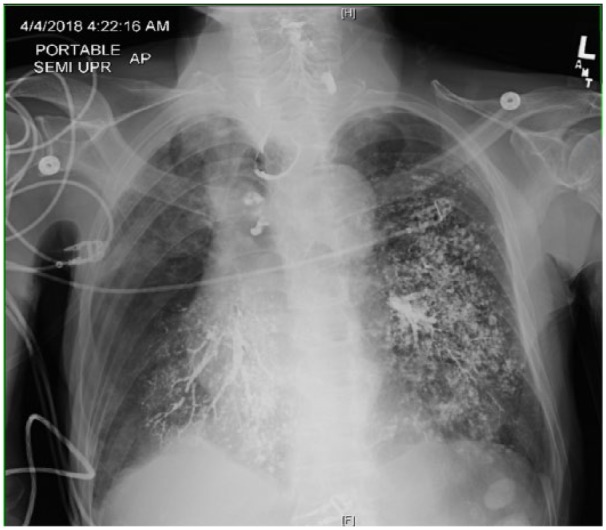
Bilateral middle and lower lobes of the lung showing barium sulfate aspiration.

**Figure 3.**
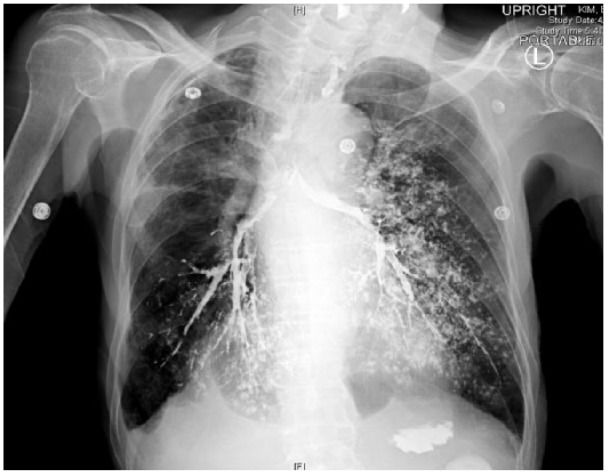
Chest X-ray showing diffuse involvement of bronchioles and alveoli more on the left side.

Chest X-ray showed contrast highlighting the tracheobronchial tree and bilateral upper lungs (Figure 2). He was managed conservatively with IV fluids and IV antibiotics. He started to improve within 6 hours of aspiration event with stabilization of vital signs including resolution of hypotension and tachycardia. Oxygen requirement also improved to 2 L through nasal cannula within 24 hours. Repeat chest X-ray showed the advancement of contrast into bilateral bronchioles and alveoli with left-sided predominance (Figure 3). His respiratory status remained stable; however, later on, due to his other comorbidities, family opted for hospice care.

## Literature Search

The available literature was systematically searched by 3 authors independently to retrieve all available material on barium sulfate aspiration. There was no language filter placed, and articles were collected from their inception till May 2018, using the MEDLINE, Cochrane, Embase, and Scopus databases. Different MeSH (Medical Subject Headings) terminologies like “barium,” “barium sulfate,” “barium sulphate,” “barium sulfate aspiration,” “barium sulphate aspiration,” “barium aspiration,” “upper gastrointestinal tract examination,” “upper gastrointestinal examination,” “barium swallow,” “upper digestive tract,” “esophageal,” “esophagus,” “gastric,” “stomach,” “duodenum,” and “duodenal” were combined using the Boolean operators “AND” and “OR” with the terms “investigation,” “examination,” swallow,” and “oral contrast.” Another author retrieved few articles through manual search using the reference list of all retrieved publications through the above-mentioned search strategy.

## Results

### Literature Retrieval and the Results

**Figure 4.**
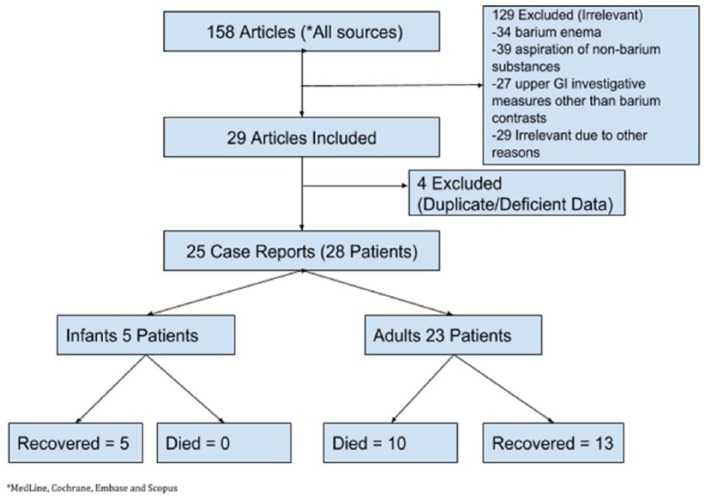
Flow sheet of literature search and prognosis of barium aspiration.

A total of 158 articles were initially obtained using the above-mentioned search strategy. The titles and abstracts of all these articles were screened for their relevance to our study, and only 29 articles were selected for full-text reading.^1-29^ A total of 129 articles were excluded as they were not related to barium sulfate aspiration, and the reasons for its exclusion are summarized in Figure 4. Of the selected 29 articles, 2 were duplicate. Hence, only o1 of these 2 articles was used in data extraction.^9,25^ Three more articles could not be obtained in full-text form and were excluded.^27-29^ As a result, a total of 25 articles were selected for the final review and analysis. The total number of patients was 28, as few cases had more than one patient (Figure 4).

### Patients Description

All these articles were published in English except one which was published in Chinese.^2^ For the total of 29 patients including ours, the number of adult patients was 23, and their mean age was 64 ± 14.52 years (range = 37-85 years). Some studies had no mention of patient age, and some had infants as patients (5 patients). Barium sulfate aspiration was found to be more common among men (18 patients) than women (11 patients). These findings are illustrated in Table 1.

**Table 1.** Characteristics of Previously Reported Cases of Barium Sulfate Aspiration.

#	Study, Year	Age/Sex	Indication	Lung(s)	Presentation	Outcome
1	Yan et al,^1^ 2017	47/female	Foreign body in esophagus	R	Asymptomatic	Recovery
2	Liu et al,^2^ 2016	45/male	Esophagopleural fistula	BL, R > L	Cough, fever	Recovery
3	Liu et al,^2^ 2016	51/male	Foreign body in esophagus	BL	Cough, fever	Recovery
4	Fuentes et al,^3^ 2014	76/male	Postoperative review	R	Asymptomatic	Recovery
5	Jackson et al,^4^ 2014	0.02/male	Gastroesophageal reflux	BL, R > L	Asymptomatic	Recovery
6	Shulan et al,^5^ 2013	N/A/female	Postoperative review	BL, R > L	Dyspnea	Mechanical ventilation
7	Gerada et al,^6^ 2013	77/male	Dysphagia	BL	Respiratory failure	Death
8	Albeldawi et al,^7^ 2012	79/male	Weight loss, dysphagia	BL, L > R	Respiratory failure	Death
9	Varatharaj et al,^8^ 2012	70/female	Dysphagia	BL	N/A	Recovery
10	Buschmann et al,^9^ 2011	64/female	Gastric cancer	BL, R > L	ARDS	Death
11	Basu et al,^10^ 2009	0.58/male	Volvulus	BL, R > L	Dyspnea	Recovery
12	Wani et al,^11^ 2008	53/male	Dysphagia	BL	Dyspnea	Recovery
13	Katsanoulas et al,^12^ 2007	37/male	Gastroesophageal reflux	BL, L > R	Asymptomatic	Recovery
14	Katsanoulas et al,^12^ 2007	43/male	Dysphagia	BL, L > R	Dyspnea, hypoxemia	Death
15	Gernez et al,^13^ 2005	66/male	Mesothelioma	BL, L > R	ARDS	Death
16	Chiu et al,^14^ 2005	1.33/female	Foreign body in esophagus	BL, R > L	ARDS	Recovery
17	Venkatraman et al,^15^ 2005	3/male	Gastroesophageal reflux	L	Mild-to-moderate ARDS	Recovery
18	Kaira et al,^16^ 2004	70/male	Gastric tumor	BL, R > L	Severe hypoxemia	Recovery
19	Fruchter et al,^17^ 2003	80/male	Dysphagia	BL	ARDS, hypotension	Death
20	Voloudaki et al,^18^ 2003	73/female	Eructation	BL, L > R	Asymptomatic	Recovery
21	Tamm et al,^19^ 1999	60/male	Esophageal tumor	BL	Hypoxemia	Death
22	Tamm et al,^19^ 1999	68/Female	Tumor recurrence	BL	ARDS	Recovery
23	Lopez et al,^20^ 1997	0.17/male	Vomiting	BL	Mild ARDS	Recovery
24	Gombar et al,^21^ 1995	48/female	N/A	L	Hypotension	Death
25	Pracy et al,^22^ 1993	60/male	Dysphagia	R	Acute pneumonia	Recovery
26	Pennington et al,^23^ 1993	81/female	Dysphagia	R	Severe complication	Recovery
27	Gray et al,^24^ 1989	85/female	Dysphagia	R	Pneumonia	Death
28	Gray et al,^24^ 1989	74/female	Dysphagia	BL, R > L	Pneumonia	Death
29	This case	84/male	Dysphagia	BL, L > R	Pneumonia	Hospice

Abbreviations: R, right; BL, bilateral; L, left; N/A, not available; ARDS, acute respiratory distress syndrome.

### Risk Factors of Barium Sulfate Aspiration

The most common risk factor for barium sulfate aspiration was found to be dysphagia (n = 11, 37.9%), followed by tumor-associated esophageal obstruction (n = 5, 17.24%) and esophageal foreign body (n = 3, 10.31%). Moreover, postoperative states, vomiting, eructation, and gastroesophageal reflux can also cause aspiration of the barium sulfate during upper gastrointestinal (GI) examination. Although more commonly reported among adults, barium aspiration should not be neglected in infants (n = 5, 17.24%) due to their high chances of aspirating the dye. Many factors like poor cooperation, coughing, and crying during the procedure most certainly increase their chances of aspiration during the examination of upper GI tract.^10,14^

### Distribution of the Aspirated Barium Sulfate in the Lungs

Barium sulfate was most commonly aspirated into bilateral lung fields (n = 22, 75.8%) followed by aspiration into the right lung (n = 5, 17.24%) with the left lung being least commonly reported (2 cases out of 29 [7.01%]).

In cases of aspiration into bilateral lung fields (n = 22), the distribution of the area affected varied; about 40% (n = 9/22) cases had equal involvement of both lungs, while 60% (n = 13/22) had either right lung 36.66% (n = 8/22) or left lung 22.7% (n = 5/22) predominance. Right lower lobe was the most commonly involved area followed by the lingual and middle lobe. Our case, however, has bilateral lung involvement with left-sided predominance, which was rarely reported (Figure 2). It is important to note that in infants the affected area was greater as compared with adults and was almost equal to the whole lung.^14,20^

### Complications of Barium Sulfate Aspiration

About 76% (22/29) of the patients had severe symptoms and complications like shortness of breath, hypoxia, respiratory failure, and acute respiratory distress syndrome (ARDS) associated with an elevated white blood count after barium sulfate aspiration. Twenty-four percent of the patients were either asymptomatic or were having mild symptoms like fever and cough.

### Concentration of Barium Sulfate and Prognosis

Only a few articles reported the exact concentration of the barium sulfate.^4,14,16,20,23,24^ The highest concentration was reported by Gray et al,^24^ which was about 250% weight/volume. This concentration led to the death of the patient described in his case. A relatively lower barium sulfate concentration (200% weight/volume) was reported in other cases.^4,14,16,20,23^ All patients in these cases survived and were discharged home with no further sequelae.

Out of the 10 patients who died, 80% (n = 8/10) required mechanical ventilation, whereas 20% (n = 2/10) died without receiving ventilatory support. The average number of days for patients who succumbed to respiratory complications was 19 days for patients who required ventilatory support and less than 24 hours for 2 patients who died without getting ventilatory support.

The survival rate among infants was 100%, while it was 56.5% (n = 13/23) in adults. Hence, the mortality among adults was high up to 43.5% (n = 10/23). About every third adult who aspirated the barium sulfate succumbed to respiratory complications. Mechanical ventilation was required in 2 of all the surviving patients including our patient.^5^

## Discussion

Barium sulfate is a heavy inorganic metal, used as a contrast agent to enhance the visualization of the GI tract by increasing the absorption of X-rays. It does not have any pharmacological activity and is neither absorbed nor metabolized by the body. It is excreted unchanged in the feces. At it is believed to be an inert substance, it does not cause any inflammatory changes in the tracheobronchial tree if aspirated. However, severe hypoxia, ARDS, and even death can happen in selected cases and can usually be attributed to factors including but not limited to simultaneous aspiration of gastric contents, anaphylactic reaction, and high-volume aspiration.^2,8,9,12,19,24,26^

High-volume barium sulfate can cause airflow obstruction due to its viscosity and the limited ability of the lungs to clear themselves.^8^ Its presence in the airspace also interferes with the gaseous exchange and causes ventilation-perfusion (V/Q) mismatch leading to hypoxemia, pneumonia, ARDS, and respiratory failure.^1^ Barium sulfate aspiration into the lungs can also have long-term sequelae like pulmonary fibrosis and bronchial granuloma.^10,18^ The severity of the airflow obstruction and respiratory complications mostly depends on the amount of barium swallowed. The normal recommended barium concentration is usually around 100 g barium sulfate in every 100 mL of preparation. Our review also showed that the extent of lung damage was directly proportional to the amount of aspirated barium sulfate. Patients with high barium concentration (250% weight/volume) died of aspiration due to respiratory complications in contrast with patients who had lower barium concentration (<200% weight/volume).^4,14,16,20,23^ Moreover, the mortality was high among patients who had barium swallow done for the evaluation of esophageal cancer.^18,24^ We hypothesize that the amount of barium aspiration in these patients was possibly high due to the downward obstruction of the gut leading to regurgitation and aspiration. Our patient, however, had no history of esophageal carcinoma, and about a 100% weight/volume barium was used and had a successful recovery.

There are no defined guidelines for the management of barium sulfate aspiration. Supportive care is usually provided after large volume barium aspiration including supplemental oxygen and chest physiotherapy to help promote clearance. Antibiotics are given due to inability to exclude bacterial superinfection.^30^ In severe cases, if the patient has been already intubated, bronchoscopy can be done to suction out barium. Bronchoalveolar lavage is controversial due to concerns about disseminated to other unaffected airways.^19^

In patients at high risk of aspiration such as elderly, history of oropharyngeal dysphagia, and head/neck malignancy, videofluoroscopic modified barium swallow (VBS) or functional endoscopic evaluation of swallowing (FEES) should be done to evaluate the swallowing mechanism before challenging with the barium bolus to look for the esophageal cause of dysphagia.^31^ Ultrasonography, endoscopy, magnetic resonance imaging, computed tomography, or positron emission tomography-computed tomography can be used as an alternative diagnostic technique.^32-37^ Second, in cases where contrast evaluation is necessary, some other lung-friendly contrast agents like iopydol, hytrast, and iodixanol can be considered.^12,19,38,39^ Last, proper posturing and maneuvers, for example, rotating the head while lying on the side and chin touching the neck during barium swallow, can prevent aspiration of barium sulfate.^40^

## Conclusion

- Oral barium sulfate aspiration is a rare but well-recognized complication of upper GI contrast-related studies. Most commonly, aspiration of barium sulfate does not lead to any clinical sequelae, but high-volume aspiration can lead to respiratory failure and circulatory shock with high mortality rate.
- Careful selection of patients for contrast-related upper GI studies should be made and if the suspicion of oropharyngeal dysphagia is high VBS or FEES should be done before esophagogram to prevent large-volume barium aspiration.
